# Dominating Cause of Pulmonary Hypertension May Change Over Time—Diagnostic and Therapeutic Considerations in a Patient with Pulmonary Hypertension Due to Rheumatoid Arthritis with Lung Involvement

**DOI:** 10.3390/diagnostics11101931

**Published:** 2021-10-19

**Authors:** Monika Szturmowicz, Monika Franczuk, Małgorzata Ewa Jędrych, Dorota Wyrostkiewicz, Karina Oniszh, Szymon Darocha, Krzysztof Kasperowicz, Marcin Kurzyna

**Affiliations:** 1 Department of Lung Diseases, National Tuberculosis and Lung Diseases Research Institute, 01-138 Warsaw, Poland; mewa.jedrych@gmail.com (M.E.J.); dw707@wp.pl (D.W.); 2 Department of Physiopathology, National Tuberculosis and Lung Diseases Research Institute, 01-138 Warsaw, Poland; monika.franczuk@gmail.com; 3 Department of Radiology, National Tuberculosis and Lung Diseases Research Institute, 01-138 Warsaw, Poland; karina.oniszh@gmail.com,; 4 Department of Pulmonary Circulation and Thromboembolic Diseases, Medical Centre of Postgraduate Medication, European Health Centre Otwock, 05-400 Otwock, Poland; szymon.darocha@ecz-otwock.pl (S.D.); krzysztof.kasperowicz@ecz-otwock.pl (K.K.); marcin.kurzyna@ecz-otwock.pl (M.K.)

**Keywords:** pulmonary arterial hypertension, rheumatoid arthritis, lung disease, right heart catheterization, body plethysmography

## Abstract

Chronic lung diseases are one of the most frequent causes of pulmonary hypertension (PH). The diagnostic challenge is to differentiate PH due to chronic lung disease from pulmonary arterial hypertension (PAH) with coexisting chronic lung disease. Moreover, the dominating cause of PH may change over time, requiring the implementation of new diagnostic procedures and new treatment modalities. We present a 68-year-old female, initially diagnosed with PH in the course of interstitial lung disease, with restrictive impairment of lung function. Therapy with immunosuppressive drugs resulted in significant clinical, radiological and functional improvement. However, five years later, arthritis symptoms developed, with PH worsening, despite stable lung disease. The patient was diagnosed with PAH in the course of rheumatoid arthritis. The introduction of sildenafil resulted in marked clinical and hemodynamic responses. Long-term survival (eleven years from PH onset and five years from PAH confirmation) has been achieved, and the patient remains in good functional condition. As the differential diagnosis of PH in patients with lung diseases is complex, the cooperation of pulmonologists and cardiologists is mandatory to obtain therapeutic success.

## 1. Introduction

Recent classification of pulmonary hypertension (PH) includes five groups: pulmonary arterial hypertension—PAH (group 1), PH due to left heart insufficiency (group 2), PH due to lung disease and/or hypoxia (group 3), PH due to pulmonary artery obstruction (group 4) and pulmonary hypertension due to miscellaneous causes (group 5) [1]. Nevertheless, in many patients, several potential reasons for PH may coexist. 

PH associated with connective tissue disease (CTD) is listed in group 1 of PH classification. When precapillary pulmonary hypertension is confirmed with right heart catheterization (RHC), it may be treated with PAH-specific drugs (RHC) [1]. In the case of lung involvement, the final diagnosis relies on the results of high-resolution computed tomography of the lungs (HRCT) and pulmonary function assessment. The extension of lung disease seen on HRCT calculated as <20% and FVC > 70% allows the exclusion of significant lung disease in PAH CTD [2]. Such criteria are used in most randomised clinical trials and are considered clinically reliable. Significant lung disease in CTD patients, resulting in PH, indicates the phenotype of group 3 PH. On such occasions, PAH-specific therapy may be applied only in the case of severe pulmonary hypertension (mPAP > 35 mmHg or mPAP ≥ 25 mmHg and CI < 2 L/min/m^2^ on RHC) [2,3,4,5]. 

We present the female patient diagnosed with PH in rheumatoid arthritis (RA) with lung involvement. The dominating cause of PH had changed in the disease, resulting in the re-classification from group 3 to group 1 of PH. A combination of immunosuppressive drugs and sildenafil (introduced after PAH confirmation) led to marked clinical improvement, with long-term survival achieved.

## 2. Case Report

**Figure 1:**
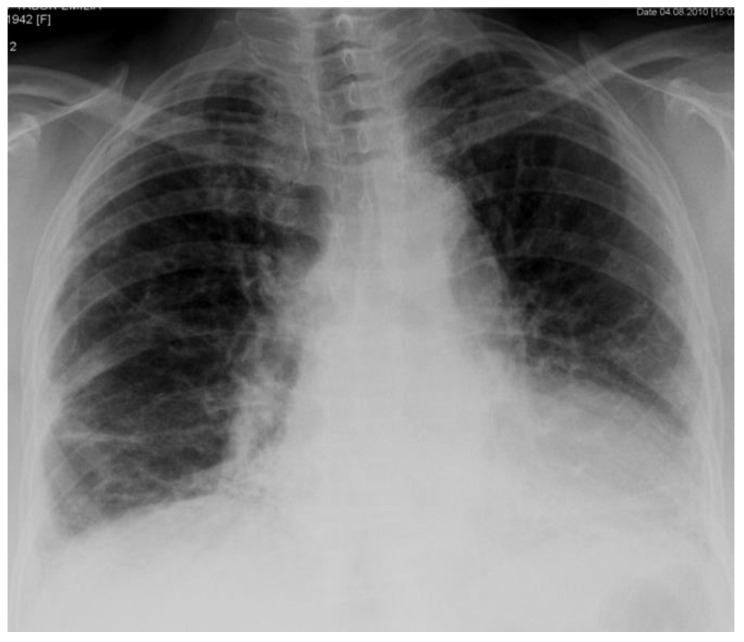
Chest X-ray (2010) shows loss of lung volume and linear opacities in the lung bases and sub-pleural region.

A 68-year-old female, diagnosed with arterial hypertension, ischemic heart disease and diabetes mellitus (type 2), was admitted to the I Department of Lung Diseases National Tuberculosis and Lung Diseases Research Institute, in August 2010 due to decreased exercise tolerance and a chronic cough, with expectoration of mucous sputum. The patient was the owner of a small farm, breeding a few hens and a cow. She did not notice the worsening of respiratory symptoms after contact with her family. She was an ex-smoker (15 pack-years). A chest X-ray revealed pulmonary abnormalities suggestive of interstitial lung disease (Figure 1). 

**Figure 2:**
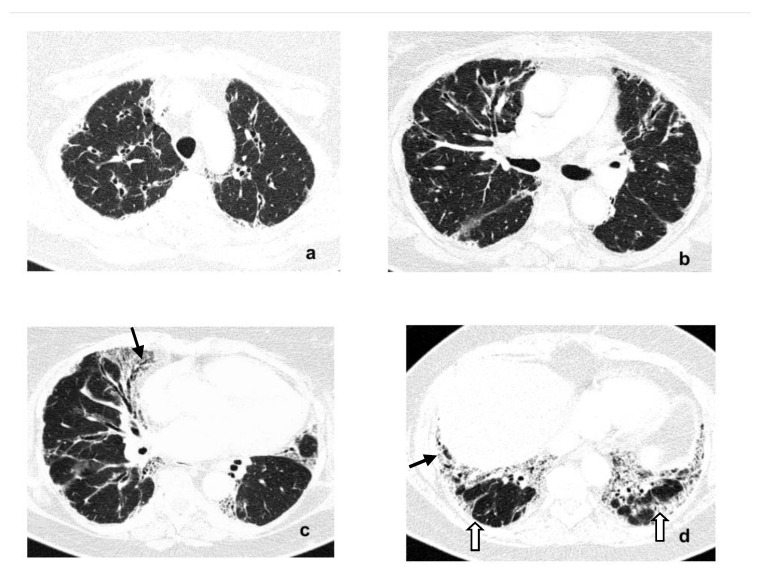
(**a**–**d**) Baseline axial high-resolution CT images (2010) on 4 selected lung levels show bilateral reticular opacities, traction bronchiectasis (**c**,**d**-black arrows) and ground-glass opacities (**d**, thick white arrows) in a basal and sub-pleural region indicating interstitial lung fibrosis.

A chest CT showed reticular opacities and traction bronchiectasis with peripheral and lower lobes predominance and coexisting small areas of ground-glass opacities (Figure 2a–d).

The usual interstitial pneumonia (UIP)-like pattern of lung fibrosis was described. The HRCT extent of lung fibrosis was estimated at 25%.

On the body plethysmography, a restrictive impairment of lung function was diagnosed (total lung capacity-TLC-53% pred.) with a severe decrease in lung transfer capacity for carbon monoxide (TLCO) value (39% pred.). However, resting oxygen saturation was within normal limits (Table 1). During the six minutes walking test (6MWT), the patient covered 346 m, with substantial desaturation 92–82%.

Echocardiographic signs of pulmonary hypertension were present, pulmonary artery systolic pressure (PASP) was 44 mmHg and pulmonary artery acceleration time (Act) was 67 ms, with a preserved left ventricle ejection fraction (LVEF) of 58%. The serum concentration of N-terminal brain natriuretic pro-peptide (NT-pro BNP) was within normal limits (Table 1). The antinuclear antibodies (ANA) titre was 1:320 with no specific ANA type found, the rheumatoid factor (RF) was 17 IU/mL (cut off < 13 IU/mL). Precipitins directed against protein antigens of birds’ droppings (pigeons, hen, ducks, parrots, turkeys) as well as antigens of thermophilic actinomycetes were not found. Fibre-optic bronchoscopy revealed the features of chronic bronchitis and the cultures of bronchial washings were negative. A lung biopsy was not performed due to decreased lung volumes and the presence of PH.

**Figure 3:**
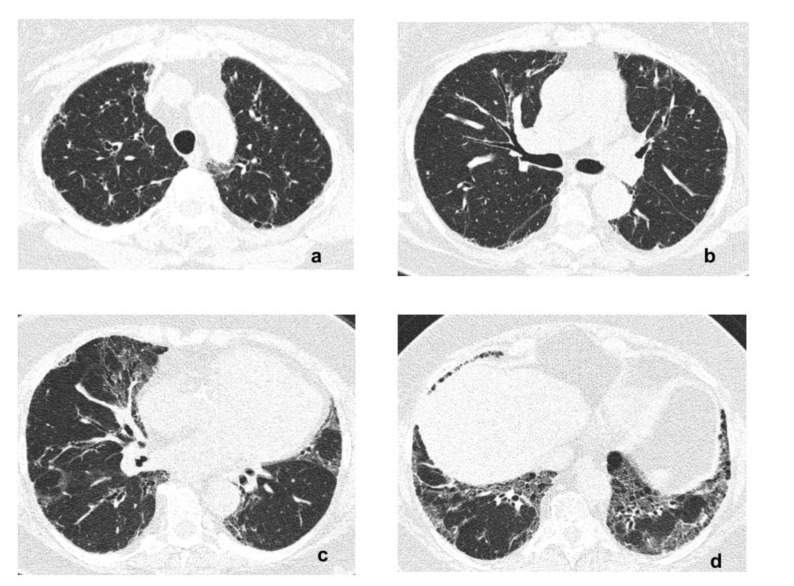
(**a**–**d**) Follow-up HRCT images (2012) show regression of ground-glass opacities and persisting reticular opacities with traction bronchiectasis.

The patient was diagnosed with PH related to lung fibrosis of an unknown origin, with signs of active pulmonary disease (ground-glass opacities). Despite the positive ANA and RF, the patient had no clinical symptoms of CTD. Due to the significant impairment of exertional lung capacity, immunosuppressive therapy with azathioprine (AZA) 150 mg/po/day and prednisone (P) 30 mg/po/day was introduced after multidisciplinary discussion. From 2010 to 2012, the patient was treated with AZA and decreasing doses of P, with marked clinical improvement. A body plethysmography revealed a significant increase in lung volume and TLCO (Table 1). Echocardiographic signs of PH diminished as well (Table 1). A follow-up chest CT scan revealed the regression of ground-glass opacities and persisting radiologic signs of lung fibrosis (Figure 3a–d); the extent of lung fibrosis was estimated at 20%.

The patient was recommended to stop AZA and further reduce the prednisone dose. In 2015, the symptomatic arthritis of small joints with morning stiffness developed, RF was increased to 110 IU/mL, and the anti-cyclic citrullinated protein antibodies (aCCP) level was 122 EU/mL (cut off < 20 EU/mL). The patient was diagnosed with rheumatoid arthritis (RA). She received leflunomide 20 mg/day for 3 months, subsequently, cyclosporine 150 mg/day for 8 months, and finally AZA 100 mg/day.

**Figure 4:**
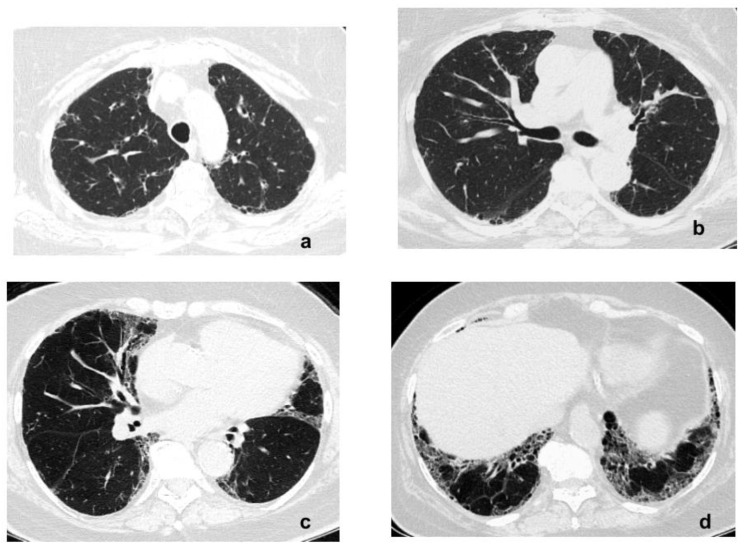
(**a**–**d**) HRCT images (2016) show no progression of interstitial lung fibrosis.

In 2016, her exercise capacity decreased again. A pulmonary function evaluation revealed a significant decrease in TLCO, but lung volumes remained within normal limits (Table 1). An echocardiography showed the progression of PH (PASP—65 mmHg), LVEF was 50%, the left atrium diameter was 36 mm, and the left ventricular end-systolic diameter was 41 mm. NT-proBNP was increased to 340 pg/mL. A chest CT angiography, performed at that time, excluded pulmonary embolism; fibrotic lung disease was not progressing (Figure 4a–d).

The patient was transferred to the Pulmonary Hypertension Centre for right heart catheterization (RHC).

RHC revealed a mean pulmonary artery pressure (mPAP) of 42 mmHg, pulmonary vascular resistance (PVR) of 5.48 Wood units (WU) and normal pulmonary capillary wedge pressure (PCWP) (Table 2).

PAH in the course of CTD was diagnosed. The REVEAL 2.0 risk score was 8 points. Subsequently, sildenafil 60 mg/day was introduced according to the National Treatment Programme of PAH. The control visits documented a marked improvement in exercise tolerance, better oxygenation during the 6MWT and that NT-proBNP decreased (Table 1). A control RHC performed 14 months later showed the normalisation of mPAP and reduction of PVR (Table 2). As a result, the REVEAL score fell to 5 points.

Eleven years from the first hospitalization, the patient is well; there is good control of lung disease and pulmonary vascular disease has been achieved.

## 3. Discussion

Rheumatoid arthritis is the most frequent CTD affecting approximately 1% of the population [6,7]. The prevalence of lung disease in RA patients is assessed at 10–68%, depending on the evaluation method [6,8].

Although RA is more common in females than males, lung involvement in RA occurs more frequently in males [9]. On the contrary, female sex is a risk factor for PAH, both idiopathic and connective tissue associated.

Lung involvement in RA concerns genetically susceptible individuals with coexisting inducing factors, such as environmental exposure to dusts or cigarette smoke [10,11]. The pathological autoantibodies directed against mucosal surfaces of the lung initiate a chronic inflammatory reaction, activating neutrophils and forming neutrophil extracellular traps (NETs) [11]. 

The most frequent type of pulmonary involvement in RA, diagnosed on HRCT, is UIP-like lung fibrosis. Non-specific interstitial pneumonia (NSIP) and organizing pneumonia (OP) is found less frequently [6,8]. Other forms of lung involvement in the course of RA, such as pulmonary nodules, pleural disease or bronchial disorders, may be diagnosed as well [8,9]. Our patient presented with a UIP-like pattern of lung fibrosis, with a small foci of ground-glass opacities.

Lung involvement may be diagnosed simultaneously with RA recognition, or it may develop in the course of the disease [11]. Occasionally, lung disease precedes RA recognition by 1–5 years [11], as the presented case shows. Male gender, smoking, increasing age and high concentrations of RF and aCCP are considered the risk factors for severe lung disease in RA [10,12]. 

The prevalence of pulmonary hypertension in the course of RA is less frequent than in scleroderma, mixed collagen tissue disease and dermatomyositis/polymyositis [2]. In the Polish National PAH Registry, RA was recognized in 11%, SSc in 48%, and MCTD in 17% of PAH CTD patients [13]. 

The differential diagnosis of PH causes in RA has to consider PAH, lung disease, left ventricular insufficiency and venous thromboembolic disease [14]. The confirmation of the dominating cause of PH implies the specific mode of therapy.

In the presented case, the initial diagnosis was PH in the course of interstitial lung disease. The interstitial lung disease was confirmed by HRCT, revealing the features of lung fibrosis. In the lung function test, a restrictive ventilatory defect (TLC—53% pred, VC—58% pred.) and low TLCO (39%) were found. At that time, CTD was not recognized.

According to recent PH guidelines, PH in the course of interstitial lung disease is diagnosed in the case of decreased forced vital capacity (FVC) to less than 70% pred. and the corresponding decrease in TLCO, as well as significant lung involvement in HRCT [5]. Goh et al. defined the criteria of considerable lung involvement in HRCT as larger than 20% [15]. Zou et al. identified the cut off levels of TLCO < 46% as indicative of exercise PH in patients with parenchymal lung diseases [16].

Immunosuppressive treatment is the standard of care in RA-ILD, but certain drugs’ efficacy is unknown [2,10]. In our patient, immunosuppressive therapy composed of AZA and P was very effective, resulting in clinical and functional improvement and regression of ground-glass opacities on HRCT. A control echocardiography documented the reduction in PH signs.

The second episode of PH worsening occurred in 2016, one year after the establishment of an RA diagnosis. The HRCT extent of lung fibrosis was calculated at 20%, lung volumes remained at 76–77% of predicted values, whereas TLCO was markedly decreased (41%). Additionally, for the first time, a serum NT-proBNP increase was noted.

Echocardiography showed progression of PH (PASP—65 mmHg) with normal left ventricular systolic function (LVEF—50%). As the patient presented risk factors for heart failure with preserved left ventricular ejection fraction, such as hypertension, coronary artery disease and type II diabetes, LV diastolic function was studied in detail. Doppler echocardiography did not show left atrial or left ventricular enlargement, neither did it show elevated left heart filling pressures. RHC confirmed the presence of PH with normal pulmonary capillary wedge pressure, excluding significant left heart failure.

The patient was diagnosed with PAH in the course of RA and treated with sildenafil. The therapy resulted in marked clinical improvement and NT-proBNP normalization. A control RHC, performed in 2018, showed the normalization of mPAP and a significant decrease in PVR. An excellent overall survival has been achieved—11 years from PH diagnosis and 5 years from the confirmation of PAH on RHC.

The recognition of PH in the course of interstitial lung disease (ILD) is burdened with a poor prognosis [17]. Negative prognostic factors in PH-ILD were identified as TLCO < 35%, 6MWT desaturation < 88%, and PVR > 4.5 WU [17]. The worst outcome is observed in IPF patients, but PH in the course of CTD with UIP-like lung involvement carries a comparably poor prognosis [10,17]. In RA-ILD patients with a UIP-like HRCT pattern, a median survival of 2.6 years was documented [10].

The prognosis in PAH CTD patients depends on the combination of risk factors included in death risk calculators, such as the REVEAL 2.0 score (13 variables) or recently with REVEAL Lite (6 parameters) [18]. Our patient had scored 8 points according to REVEAL 2.0 at diagnosis; thus, the risk of death in 1 year was moderate (5–10%). However, at restaging, she received 5 points. Therefore, the risk of death decreased to <5% in 1 year.

Coexisting lung disease worsens the prognosis in PAH CTD [19], but it is also a bad prognostic factor in IPAH, especially for the 5-years perspective [20]. 

One of the reasons for the worse life expectancy in PAH patients with coexisting lung disease is the mixed origin of pulmonary vasculopathy, and it may partly depend on hypoxic pulmonary vasoconstriction. Vasodilatation in the course of PAH-specific therapy may increase the blood flow in poorly ventilated areas, increasing right to left shunt [4]. On the other hand, PAH-specific drugs lower pulmonary vascular resistance, reducing right heart insufficiency and increasing the left ventricular output. In consequence, that treatment should improve exercise capacity, resulting in less desaturation on exertion. Such a mechanism was proven in COPD patients by Blanco et al., who observed that a single dose of sildenafil decreased both mPAP and PaO_2_ during rest but during exercise, PaO_2_ was stable [21].

The clinical effect of PAH-specific drugs in patients with lung disease coexisting with PAH depends on the extent of the lung disease, degree of right ventricular insufficiency, and the class of the drug used. Previous studies of Zisman et al. and Han et al. (STEP IPF and subgroups analysis) showed no adverse influence of sildenafil on oxygenation in IPF patients [22,23]. Therefore, most PH-LD patients included in PH registries receive phosphodiesterase 5 (PDE 5) inhibitors [24,25,26]. More recently, a randomized clinical trial with inhaled treprostinil demonstrated a significant improvement in exercise capacity in patients with PH in the course of various ILDs (IPF, combined pulmonary fibrosis and emphysema, and CTD-ILD), without the decrease in oxygenation [27]. Our patient received sildenafil with good clinical and haemodynamic effects. No worsening of hypoxemia was observed.

In summary, the presented case of RA-associated PH illustrates the complexity of PH causes in a single individual—PH-ILD dominated in the early observation time, whereas PAH CTD developed in the later disease course. The multi-speciality approach of pulmonologists and cardiologists resulted in a precise diagnosis and successful therapy with immunosuppressive drugs and sildenafil as the add-on therapy. As a result, good control of both interstitial lung disease and pulmonary vascular disease, as well as long-term survival, have been achieved.

## Figures and Tables

**Table 1:** Body plethysmography, TLCO, resting oxygen saturation, 6 minutes walking test, serum NT-proBNP and echocardiography were performed between 2010 and 2021. PFT values were calculated according to GLI reference values for spirometry, TLCO and lung volumes (http://gli-calculator.ersnet.org, accessed on 20 August 2021).

Parameter/Year of Assessment	2010	2012	2016	2018	2021
TLC% pred. VC% pred. TLCO% pred.	53 58 39	71 73 56	77 76 41	79 81 61	86 79 58
Resting SaO_2_%	92	95	94	94	93
NT-proBNP pg/mL	72	56	340	116	77
6MWT (meters, desaturation%)	346 92–82	588 95–86	460 94–89	414 97–91	442 95–89
PASP mmHg Act ms	44 67	35 100	65 68	39 81	na na

TLC—total lung capacity, VC—vital capacity, TLCO—lung transfer capacity for carbon monoxide, SaO2—oxygen saturation, NT-proBNP—N-terminal brain natriuretic pro-peptide, 6MWT—six minutes walking test, PASP—pulmonary artery systolic pressure, Act—pulmonary artery acceleration time, PFT—pulmonary function tests, na—not assessed.

**Table 2:** Results of right heart catheterization performed from 2016 to 2020.

Parameter/Year of Assessment	2016	2018	2020
mPAP (mmHg)	42	23	29
mRAP (mmHg)	3	5	8
PCWP (mmHg)	10	9	11
CI (L/min/m^2^)	3.22	2.73	3.42

mPAP—mean pulmonary artery pressure, mRAP—mean right atrial pressure, PCWP—pulmonary capillary wedge pressure, CI—cardiac index.

## Data Availability

The clinical data of the patient are available in hospital database.

## References

[1] Simonneau G., Montani D., Celermajer D.S., Denton C.P., Gazoulis M.A., Krowka M., Williams P.G., Souza R. (2019). Haemodynamic definitions and updated clinical classification of pulmonary hypertension. Eur. Respir. J..

[2] Fayed H., Coglan J.G. (2019). Pulmonary hypertension associated with connective tissue disease. Semin. Respir. Crit. Care Med..

[3] Dweik R.A., Rounds S., Erzurum S.C., Archer S., Fagan K., Hassoun P.M., Hill N.S., Humbert M., Kawut S.M., Krowka M. (2014). An official American Thoracic Society statement: Pulmonary hypertension phenotypes. Am. J. Respir. Crit. Care Med..

[4] Olschewski H. (2021). The challenge to decide between pulmonary hypertension due to chronic lung disease and PAH with chronic lung disease. Diagnostics.

[5] Nathan S.D., Barbera J.A., Gaine S.P., Harari S., Martinez F.J., Olschewski H., Olsson K.M., Peacock A.J., Pepke-Zaba J., Provencher S. (2019). Pulmonary hypertension in chronic lung disease and hypoxia. Eur. Respir. J..

[6] Fischer A., Cosgrove G.P. (2014). Interstitial lung abnormalities in rheumatoid arthritis are common and important. Chest.

[7] Mathai S.C., Danoff S.K. (2016). Management of interstitial lung disease associated with connective tissue disease. Brit. Med. J..

[8] Ciancio N., Pavone M., Torrisi S.E., Vanchieri A., Sambataro D., Palmucci S., Vanchieri C., Di Marco F., Sambataro G. (2019). Contribution of pulmonary function tests (PFTs) to the diagnosis and follow up of connective tissue diseases. Multidiscip. Respir. Med..

[9] Shaw M., Collins B.F., Ho L.A., Rhagu G. (2015). Rheumatoid arthritis-associated lung disease. Eur. Respir. Rev..

[10] Matson S., Lee J., Eickelberg O. (2021). Two sides of the same coin? A review of the similarities and differences between idiopathic pulmonary fibrosis and rheumatoid arthritis-associated interstitial lung disease. Eur. Respir. J..

[11] McDermott G.C., Doyle T.J., Sparks J.A. (2021). Interstitial lung disease throughout the rheumatoid arthritis disease course. Curr. Opin. Rheumatol..

[12] Doyle T.J., Dellaripa P.F., Batra K., Frits M.L., Iannaccone C.K., Hatabu H., Nishino M., Weinblatt M.E., Asherman D.P., Washko G.R. (2014). Functional impact of a spectrum of interstitial lung abnormalities in rheumatoid arthritis. Chest.

[13] Kopeć G., Kurzyna M., Mroczek E., Chrzanowski Ł., Mularek-Kubzdela T., Skoczylas I., Kuśmierczyk B., Pruszczyk P., Błaszczak P., Lewicka E. (2020). Characterization of patients with pulmonary arterial hypertension: Data from Polish Registry of Pulmonary Hypertension (BNP-PL). J. Clin. Med..

[14] Panagiotidou E., Sourla E., Kotoulas S.X., Akritidou S., Bikos V., Bagalas V., Stanopoulos I., Pitsiou G. (2017). Rheumatoid arthritis-associated pulmonary hypertension: Clinical challenges reflecting the diversity of pathophysiology. Respir. Med. Case Rep..

[15] Goh N.S., Desai S.R., Veeraraghavan S., Hansell D.M., Copley S.J., Maher T.M., Corte T.J., Sander C.R., Ratoff J., Devaraj A. (2008). Interstitial lung disease in systemic sclerosis: A simple staging system. Am. J. Respir. Crit. Care Med..

[16] Zou R.H., Wallace W.D., Nouraie S.M., Chan S.Y., Risbano M.G. (2020). Lower DLCO% identifies exercise pulmonary hypertension in patients with parenchymal lung disease referred for dyspnoea. Pulm. Circ..

[17] Alhamad E.H., Cal J.G., Alrajhi N.N., Alharbi W.M. (2020). Predictors of mortality in interstitial lung disease-associated pulmonary hypertension. J. Clin. Med..

[18] Benza R.L., Kanwar M.K., Raina A., Scott J.V., Zhao C.L., Selej M., Elliot G., Farber H.W. (2021). Development and validation of an abridged version of the REVEAL 2.0 risk score calculator, REVEAL Lite 2, for use in patients with pulmonary arterial hypertension. Chest.

[19] Chauvelot L., Gamondes D., Berthiller J., Nieves A., Renard S., Catella-Chatron J., Ahmad K., Bertoletti L., Camara B., Gomez E. (2020). Hemodynamic response to treatment and outcome in pulmonary hypertension associated with interstitial lung disease versus pulmonary arterial hypertension in systemic sclerosis. Arth. Rheum..

[20] Lewis R.A., Thompson A.A.R., Billings C.G., Charalampopoulos A., Elliot C.A., Hamilton N., Hill C., Hurdman J., Rajaram S., Sabroe I. (2020). Mild parenchymal lung disease and/or low diffusion capacity impacts survival and treatment response in patients diagnosed with idiopathic pulmonary arterial hypertension. Eur. Respir. J..

[21] Blanco I., Gimeno E., Munoz P.A., Pizarro S., Gistau C., Rodriguez-Roisin R., Roca J., Barbera J.A. (2010). Hemodynamic and gas exchange effects of sildenafil in patients with chronic obstructive pulmonary disease and pulmonary hypertension. Am. J. Respir. Crit. Care Med..

[22] Zisman D.A., Schwarz M., Anstrom K.J., Collard H.R., Flaherty K.R., Hunninghake G.W. (2010). A controlled trial of sildenafil in advanced idiopathic pulmonary fibrosis. N. Engl. J. Med..

[23] Han M.K., Bach D.S., Hagan P.G., Yow E., Flaherty K.R., Toews G.B., Anstrom K.J., Martinez F.J., IPFnet Investigators (2013). Sildenafil preserves exercise capacity in patients with idiopathic pulmonary fibrosis and right-sided ventricular dysfunction. Chest.

[24] Hoeper M.M., Behr J., Held M., Grunig E., Vizza C.D., Vonk-Noordegraaf A., Lange T.J., Claussen M., Grohe C., Klose H. (2015). Pulmonary hypertension in patients with chronic fibrosing idiopathic interstitial pneumonias. PLoS ONE.

[25] Brewis M.J., Church A.C., Johnson M.K., Peacock A.J. (2015). Severe pulmonary hypertension in lung disease: Phenotypes and response to treatment. Eur. Respir. J..

[26] Tanabe N., Kumamaru H., Tamura Y., Taniguchi H., Emoto N., Yamada Y., Nishiyama O., Tsujino I., Kuraishi H., Nishimura Y. (2021). Multi-institutional prospective cohort study of patients with pulmonary hypertension associated with respiratory diseases. Circ. J..

[27] Waxman A., Restrepo-Jaramillo R., Thenappan T., Ravichandran A., Engel P., Bajwa A., Allen R., Feldman J., Argula R., Smith P. (2021). Inhaled treprostinil in pulmonary hypertension due to interstitial lung disease. N. Engl. J. Med..

